# Validation of the Arabic Version of the Diabetes Therapy-Related Quality of Life Questionnaire (DTR-QOL) in Tabuk, Saudi Arabia: A Pilot Study

**DOI:** 10.7759/cureus.69805

**Published:** 2024-09-20

**Authors:** Hisham Alshadfan, Hyder Mirghani, Tariq Alrasheed, Yassin Ibrahim, Samar Aljohani, Tahani Alanzi, Sami Alshehri, Armania Nurdin, Muhammad Nazrul Hakim Abdullah

**Affiliations:** 1 Department of Biomedical Sciences, Faculty of Medicine and Health Sciences, Universiti Putra Malaysia, Serdang, MYS; 2 Department of Clinical Biochemistry, Faculty of Medicine, University of Tabuk, Tabuk, SAU; 3 Department of Internal Medicine, Faculty of Medicine, University of Tabuk, Tabuk, SAU; 4 Department of Population Health Management, Tabuk Health Cluster, Ministry of Health, Tabuk, SAU; 5 Department of Family and Community Medicine, Faculty of Medicine, University of Tabuk, Tabuk, SAU; 6 Department of Medical Laboratory, King Fahad Specialist Hospital, Ministry of Health, Tabuk, SAU

**Keywords:** diabetes mellitus, diabetes therapy-related quality of life questionnaire, internal consistency, reliability, validity

## Abstract

Background: Diabetes mellitus is a chronic disease that significantly influences the patient's quality of life. This serious disease is approaching an epidemic, and the Arab countries are in the diabetes super region. Improving the patient's quality of life is vital for good glycemic control. However, the Diabetes Therapy-Related Quality of Life Questionnaire (DTR-QOL) is not available in Arabic. Thus, we aimed to validate the questionnaire among patients with diabetes in Saudi Arabia.

Subjects and methods: This pilot study was conducted among randomly selected 30 patients with diabetes who came for regular follow-up in the Diabetes Center in King Fahd Specialist Hospital in Tabuk City, Saudi Arabia, during March and April 2023. A structured questionnaire based on sociodemographic data and the DTR-QOL was used. The information collected were age, gender, diabetes medications, glycemic indices, lipid profile, fasting insulin, homeostatic model assessment for insulin resistance, and body mass index. Forward/backward translation, expert assessment, and Cronbach's alpha were used to assess the validity and reliability.

Results: Out of the 30 patients with diabetes piloted for the questionnaire validation, 60% were females, the mean age was 51.1 ± 14.056 years, and 94% had type 2 diabetes. The internal consistency varied from 0.80 for hypoglycemia to 0.94 for anxiety and dissatisfaction with treatment. The Arabic version of the DTR-QOL is valid and reliable for use among patients with diabetes, with a content validity of 0.938 and Cronbach's alpha of 0.93.

Conclusion: The Arabic version of the DTR-QOL is valid and reliable for use among patients with diabetes in Arab countries and has good sensitivity and consistency.

## Introduction

The diabetes mellitus epidemic is ongoing, and the projection for the age-standard prevalence is >10% globally by the year 2050. The Arab world lies in the diabetes super region (Middle East and North Africa) with an expected prevalence of 16.8% [1].

Diabetes mellitus poses a negative impact on the quality of life with increasing morbidity and mortality; therefore, assessing and improving the quality of life is at the center of chronic disease management, including diabetes [2,3]. Because diabetes mellitus is a chronic disease with short and long-term grave consequences and no cure is available, it is imperative to adopt clinical measures to improve glycemic control strictly. However, the ultimate goal is to prevent deterioration and improve the patient's quality of life [4]. Quality of life includes emotional, physical, and social factors, and all are negatively influenced by diabetes because patients with diabetes are under significant pressure to manage themselves [5]. An essential component of quality of life among patients with diabetes is therapy-related (motivation and adherence to therapy). Low motivation/adherence to diabetes therapy is the main factor that increases the patient's morbidity and mortality due to poor glycemic control and poor quality of life [6]. Because new diabetes drugs are discovered regularly and many are on the pipe, the development, validation, and translation of the Diabetes Therapy-Related Quality of Life Questionnaire (DTR-QOL) is important [7,2].

The DTR-QOL consists of closed-ended questions that exhaustively include all the possible answers expected from the respondents. The English versions of the questionnaires have been previously validated for use among patients with diabetes mellitus with good reliability and validity. They can evaluate the impact of diabetes treatment on patient quality of life. The DTR-QOL can be used in any treatment method that patients have. This characteristic facilitates detecting a difference in patients' quality of life between treatment methods before and after a treatment change [8]. The current pilot study aimed to translate the English version of the DTR-QOL into the Arabic language and assess its validity among patients with diabetes.

## Materials and methods

Subjects and methods

This pilot study was conducted among randomly selected 30 patients with diabetes who came for regular follow-up in the Diabetes Center in King Fahd Specialist Hospital in Tabuk City, Saudi Arabia, during the period from March to April 2023. A systematic random sampling technique was used to select the respondents. The Diabetes Center was selected because it is the main center for diabetes care in Tabuk City and serves about 5000 patients with diabetes monthly.

Inclusion criteria

The inclusion criteria for this study were designed to select a well-defined and relevant group of respondents. Eligible respondents must be adults aged 18 years and older, have a confirmed diagnosis of diabetes mellitus, and be undergoing regular diabetes treatment, including medication or insulin therapy. Additionally, respondents must be fluent in Arabic to ensure they can fully understand and accurately complete the Arabic version of the questionnaire, which is crucial for the validity and reliability of the data. Furthermore, they must regularly attend follow-up visits at the Diabetes Center in King Fahd Specialist Hospital, Tabuk, Saudi Arabia, during the study period to ensure consistent care and monitoring, allowing for an accurate assessment of the questionnaire's applicability across different clinical interactions.

Exclusion criteria

The exclusion criteria for this study encompass several key factors to ensure a targeted and relevant sample. Firstly, individuals who do not have a confirmed diagnosis of diabetes mellitus are excluded, as the study specifically targets patients with diabetes. Secondly, individuals with cognitive impairments or psychiatric disorders that hinder their ability to understand and complete the questionnaire are also excluded. Also, pregnant women with gestational diabetes or any pregnancy-related complications are excluded to maintain consistency with the study's focus on chronic diabetes conditions. Additionally, the study is limited to Arabic-speaking respondents due to the use of an Arabic version of the questionnaire; therefore, individuals who are not Arabic speakers are excluded due to language barriers. Moreover, any individual unwilling to participate or unable to provide informed consent is excluded from the study. Finally, patients who do not consistently attend follow-up visits at the Diabetes Center are excluded, as regular follow-up is crucial for accurate data collection and study outcomes.

Sample size rationale

This pilot study aimed to validate the Arabic version of the DTR-QOL. The primary goal is to assess the feasibility, validity, and reliability of the questionnaire rather than making definitive conclusions that require larger sample sizes.

The rationale for the sample size in this pilot study is addressed regarding the nature of the pilot study, which typically involves a smaller sample size. Pilot studies are preliminary investigations designed to test the feasibility of the study design, tools, and methods before launching a larger, more definitive study. The sample included male and female respondents, ensuring a level of diversity within the sample, which is necessary to test the questionnaire's applicability across different subgroups within the population [9,10].

In studies focusing on the validation of questionnaires, sample sizes of 20 to 50 respondents are commonly used. This range is generally considered sufficient to test the questionnaire's reliability (e.g., internal consistency using Cronbach's alpha) and to make initial validity assessments. The sample size of 30 respondents in this study is appropriate for a pilot study focused on the initial validation of a questionnaire. It allows the researchers to assess content validity, face validity, and internal consistency using Cronbach’s alpha. Pilot studies usually use smaller samples, allowing researchers to make preliminary observations and refine methods for larger studies [11,12].

Measures

A structured questionnaire based on sociodemographic data (SBL-R) and the Oral Hypoglycemic Agent Questionnaire (OHA-Q) version 2 were used. The information in SBL-R included the duration of diabetes, gender, age, current diabetes medications, body measurements, glycemic indices, lipid profile, fasting insulin, and homeostatic model assessment for insulin resistance. The DTR-QOL includes 29 items distributed in four domains: burden on social and daily activities (BSDA), which comprises 13 items; anxiety and dissatisfaction with treatment (ADT), which contains eight items; hypoglycemia (HG), which consists of four items; and satisfaction with treatment (ST), which includes four items. The response scale was a seven-point Likert scale (1: strongly agree; 7: strongly disagree). The score of each item was reversed (7 represents the highest quality of life, and 1 represents the lowest quality of life). The total score was calculated from the mean score for all items and converted to 0-100 (best response = 100; worst response = 0) [8].

Ethical clearance

All respondents signed written informed consent, and the research was ethically cleared by the ethical committee of the University of Tabuk (Ref.: UT-190-46-2022; date: March 16, 2022), the ethical committee of the Ministry of Health (Saudi Arabia) (Ref.: TU-077/022/137; date: June 14, 2023), and the ethical committee of the Universiti Putra Malaysia (Ref.: JKEUPM-2022-860; date: March 7, 2023).

Statistical analysis

SPSS version 29 (IBM Corp., Armonk, NY) was used for data analysis. The tests used for questionnaire validity were content validity, face validity, and Cronbach’s alpha. A p-value of <0.05 was considered significant.

## Results

Content validity

Content validity aimed to take all the procedures to make the items used in this questionnaire relevant and cover all the aspects of the studied issues. These include suggestions for the appropriate measurement tool and identifying and treating all sources of ambiguous words, errors, jargon, double-barreled phrases, and other vague technical terms before questionnaire administration.

In preparing a multi-item questionnaire, the content validity may be examined with an expert panel, focus groups, or in-depth interviews with respondents [11]. Content review was carried out for the English version by a panel of four experts with adequate knowledge and experience in the field of study: a professor of internal medicine and endocrine, two assistant professors of family medicine, and a consultant in epidemiology and biostatistics, at the Faculty of Medicine, University of Tabuk, Tabuk, Saudi Arabia. Then, the questions and phrases used in the questionnaire were checked by English language experts from the Institute of English Language, University of Tabuk, Tabuk, Saudi Arabia. Their valuable comments and corrections were considered, and the appropriate action was taken. The experts evaluated the questionnaire according to four items (relevance, clarity, simplicity, and ambiguity). Their opinion was given a score according to the evaluation guide, ranging from 1 to 4 for each item. If the expert score regarding an item is ≤2, it will be considered as “disagree”; and if the score is ≥3, it will be considered as “agree,” as illustrated in Table 1. The results of the panel of experts are provided in Tables 2, 3.

**Table 1:** Content of the experts’ evaluation.

Score	Relevance	Clarity	Simplicity	Ambiguity
1	Not relevant	Not clear	Not simple	Doubtful
2	The item needs some revision	The item needs some revision	The item needs some revision	The item needs some revision
3	Relevant, but it needs some revision	Clear, but it needs minor revision	Simple, but it needs minor revision	No doubt, but it needs some revision
4	Very relevant	Very clear	Very simple	Meaning is clear

**Table 2:** Content validity for SBL-R. SBL-R: structured questionnaire based on sociodemographic data.

Criteria	Expert 1	Expert 2	Expert 3	Expert 4	No. of agreements
Relevance	Agree	Agree	Agree	Agree	4
Clarity	Agree	Agree	Agree	Agree	4
Simplicity	Agree	Agree	Agree	Agree	4
Ambiguity	Agree	Agree	Agree	Agree	4
Overall experts	1	1	1	1	
Content validity index	1				

**Table 3:** Content validity for DTR-QOL. DTR-QOL: Diabetes Therapy-Related Quality of Life Questionnaire.

Criteria	Expert 1	Expert 2	Expert 3	Expert 4	No. of agreements
Relevance	Agree	Agree	Agree	Agree	4
Clarity	Agree	Agree	Agree	Agree	4
Simplicity	Agree	Agree	Agree	Agree	4
Ambiguity	Agree	Disagree	Agree	Agree	3
Overall experts	1	0.75	1	1	
Content validity index	0.938				

Our findings revealed that the content validity index for SBL-R and DTR-QOL were 1 and 0.938, respectively, suggesting that this instrument - in English form - is a valid tool for use among patients with diabetes in Arab countries [13].

Translation of the questionnaire

Since only an English questionnaire has been available, and all subjects of our sample in the Diabetes Center are Arabic speakers, the questionnaire was translated into a simplified Arabic language by trusted experts in translation from the Institute of English Language, University of Tabuk, Tabuk, Saudi Arabia. It was then translated back to English by other translation experts to ensure that the meaning was still the same in both English and Arabic versions. The objective of translation was achieved. The English and Arabic versions of the questionnaire are attached as appendices.

Face validity

The face validity was assessed by giving the questionnaire to the same population of the studied subjects to go through as a part of the pre-testing, which aims to discover any problem that might appear throughout the distribution and answering of the questionnaire. This test helps validate the questions and ensures that the respondents easily understand and answer all items in the Arabic form of the questionnaire.

Besides the abovementioned purposes, the pre-testing was also done to assess the questionnaire’s clarity, readability, and cultural sensitivity. Pre-testing was also performed to observe the feasibility and acceptability of the questionnaire, as well as the time needed for the direct face-to-face interview sessions with each respondent to complete the questionnaire.

The pre-testing was done in the Diabetes Center, King Fahad Specialist Hospital, Tabuk, Saudi Arabia. A consecutive convenience sample of 15 responsive, cooperative, and talkative respondents was selected, and they were advised to feel free to ask if they could not understand any question or word or if any item needed further explanation. The questionnaire was administered to those 15 respondents through direct face-to-face interviews. After each questionnaire administration, items were subjected to revision and correction of problematic questions according to the respondents’ inquiries and comments. Then, the procedure was applied to the second respondent, and so forth. After the fourth respondent, it was found that the respondents fully understood the questionnaire items without any further comments.

In the beginning, it was found that the time taken to interview one respondent was quite long, but fortunately, the time decreased with the following interviews. The estimated average and the minimum and maximum time needed by each interviewer and respondent to complete a single face-to-face interview during pre-testing were 15 minutes and 10 to 20 minutes, respectively.

Reliability measurements

The reliability testing for the questionnaire was done to ensure that the measurements obtained in one sitting were representative and stable over time. The significant goal of the pilot study was to collect data that could be used to test the validity and reliability of the questionnaire’s items. For the result not to be faulty, selecting a heterogeneous group of respondents with good variations was needed. For the test-retest reliability test, each respondent interviewed was requested to come back again a week later, with a maximum of four weeks after the first interview to be re-interviewed again by the same first interviewer.

A sample of 30 respondents was selected to participate in this pilot study. Respondents were recruited through a consecutive sampling by taking every person willing to participate until the sample size for the pilot study was achieved. Representation of males and females was managed to be equal in the sample. A total of 30 respondents completed the first interview and were all requested voluntarily to come back for re-interviewing (some of them came back spontaneously and some after they were called through the phone). Only 28 (93.33%) persons responded and came back for the re-interviewing, and the majority of them were females 18 (64.29%). Respondents' median and range of time to come back for re-interview were 22 days and nine to 27 days, respectively.

Reliability measurement for DTR-QOL

The internal consistency reliability of DTR-QOL was assessed using Cronbach's alpha coefficient, which is expressed as a number between 0 and 1, and values above 0.7 are acceptable [12]. It was found that the overall Cronbach's alpha for DTR-QOL was 0.93 (Table 4). Thus, the internal consistency of DTR-QOL was reliable for use among patients with diabetes in Arab countries.

**Table 4:** Internal consistency of DTR-QOL. DTR-QOL: Diabetes Therapy-Related Quality of Life Questionnaire.

Domain/subscale	No. of items	Cronbach’s alpha
Burden on social and daily activities (BSDA)	13	0.92
Anxiety and dissatisfaction with treatment (ADT)	8	0.94
Hypoglycemia (HG)	4	0.80
Satisfaction with treatment (ST)	4	0.89
Overall Cronbach’s alpha	29	0.93

## Discussion

Diabetes mellitus negatively impacts the patient's quality of life, and diabetes-specific quality of life was associated with poor glycemic control, indicating the importance of evaluating the various parameters of quality of life, including therapy-related [14]. The goal of diabetes treatment is to maintain the patient's quality of life and reduce mortality by reducing both diabetes microvascular and macrovascular complications. Good treatment satisfaction and medication adherence are predictors of physical and mental health [15,16]. An important issue is the patients' satisfaction with their treatment due to the impact on adherence and quality of life [16]. In the State of Qatar, Wilbur et al. validated the Arabic version of the diabetes treatment satisfaction questionnaire; the study was conducted among 100 patients with diabetes (mean age = 50.7 years; 54% women). Interestingly, women were less satisfied with their treatment, which may affect their adherence to medications, high glycemic control, and poor quality of life [17]. The DTR-QOL was developed with good reliability and validity to measure the effects of various diabetes therapies on quality of life [8]. However, it is not available in Arabic. There are 55 million people with diabetes mellitus in the Middle East and North Africa, and the Arabic language is used by the majority [18]. Therefore, validation of an Arabic version to assess the DTR-QOL is justifiable. There are many useful questionnaires for diabetes therapy-related quality of life, but the differences between the Arab population and other people in other countries call for an Arabic language version of the questionnaire [19]. Another justification is that the prescription pattern could be different from other parts of the world. In developing countries, glucagon-like peptide-1 (GLP-1) agonists, in particular, oral formulations, are not widely prescribed due to unavailability and cost. Moreover, patients with poor glycemic control are usually prescribed insulin [20]. Importantly, patients with needle phobia and some physicians could avoid multiple injections [21]. The Arabic version of the questionnaire was valid and reliable in line with the original version and similar to it because Ishii's [8] original version was limited by the lower representation of patients with type 1 diabetes. In addition, novel drugs with cardiac and renal protection and long half-life, including GLP-1 agonists and sodium-glucose transporters inhibitors, could positively influence the patient's quality of life. Furthermore, some long-acting drugs might not be licensed at the time of the original version [22].

Study limitations

The relatively small number of piloted patients and the reliance on a self-administered questionnaire, which is more prone to subjectivity, limited this study.

## Conclusions

Based on the excellent content validity as evaluated by four experts in medicine and biostatistics, the reliability process of the sociodemographic section of the DTR-QOL, and the very good internal consistency of all questionnaire’s domains, the Arabic version of the DTR-QOL is valid and reliable for use among patients with diabetes in Arab countries with good sensitivity and consistency.

## Appendices

DTS-QOL - English version

**Table 5:** Diabetes Therapy-Related Quality of Life Questionnaire (DTS-QOL). 1: strongly agree; 7: strongly disagree.

Diabetes Therapy-Related Quality of Life Questionnaire (DTS-QOL)	
Respondent’s Code: ………………………………………	
Domain 1: Burden on social and daily activities	
Q1	My current diabetes treatment interferes with my work and activities.
	1 2 3 4 5 6 7
Q2	My current diabetes treatment limits the scope of my activities.
	1 2 3 4 5 6 7
Q3	It is difficult to find places on time for my current diabetes treatment.
	1 2 3 4 5 6 7
Q4	My current diabetes treatment interferes with group activities and personal friendships.
	1 2 3 4 5 6 7
Q5	It is a burden getting up at a certain time every morning for my current diabetes treatment.
	1 2 3 4 5 6 7
Q6	With my current diabetes treatment, the restricted meal times are a burden.
	1 2 3 4 5 6 7
Q7	When I eat out, it is difficult to manage my current diabetes treatment.
	1 2 3 4 5 6 7
Q8	I feel like my current diabetes treatment takes away the enjoyment of eating.
	1 2 3 4 5 6 7
Q9	With my current diabetes treatment, it is hard to curb my appetite.
	1 2 3 4 5 6 7
Q10	The time and effort to manage my current diabetes treatment are a burden.
	1 2 3 4 5 6 7
Q11	I am constantly concerned about the time to manage my current diabetes treatment.
	1 2 3 4 5 6 7
Q12	Pain due to my current diabetes treatment is uncomfortable.
	1 2 3 4 5 6 7
Q13	Gastrointestinal symptoms (nausea, passing gas, diarrhea, abdominal pain) due to my current diabetes treatment are uncomfortable.
	1 2 3 4 5 6 7
Domain 2: Anxiety and dissatisfaction with treatment	
Q14	I am bothered by weight gain with my current diabetes treatment.
	1 2 3 4 5 6 7
Q15	I have uncomfortable symptoms due to hyperglycemia (high blood glucose).
	1 2 3 4 5 6 7
Q16	I am worried about high blood glucose.
	1 2 3 4 5 6 7
Q17	I am dissatisfied that my blood glucose is unstable (high and low).
	1 2 3 4 5 6 7
Q18	I am worried that complications might get worse with my current diabetes treatment.
	1 2 3 4 5 6 7
Q19	I got anxious thinking about living while on my current diabetes treatment.
	1 2 3 4 5 6 7
Q20	I find it unbearable to think that even if I continue my current diabetes treatment, my diabetes may not be cured.
	1 2 3 4 5 6 7
Q21	I am concerned that if I continue my current diabetes treatment, the efficacy (effectiveness) may diminish.
	1 2 3 4 5 6 7
Domain 3: Hypoglycemia	
Q22	I worry about low blood glucose due to my current diabetes treatment.
	1 2 3 4 5 6 7
Q23	I am scared because of low blood glucose.
	1 2 3 4 5 6 7
Q24	I am sometimes bothered by low blood glucose.
	1 2 3 4 5 6 7
Q25	Symptoms due to low blood glucose are uncomfortable.
	1 2 3 4 5 6 7
Domain 4: Satisfaction with treatment	
Q26	Overall, I am satisfied with my current blood sugar control (glycemic control).
	1 2 3 4 5 6 7
Q27	With my current diabetes treatment, I am confident that I can maintain good blood glucose control.
	1 2 3 4 5 6 7
Q28	I am hopeful about the future with my current diabetes treatment.
	1 2 3 4 5 6 7
Q29	With regard to diabetes treatment, I am satisfied with the current treatment methods.
	1 2 3 4 5 6 7

 DTS-QOL - Arabic version

**Table 6:** (DTS-QOL) استبيان علاقة علاج مرض السكري بالمستوى المعيشي (1: موافق بشدة؛ 7: غير موافق بشدة)

(DTS-QOL) استبيان علاقة علاج مرض السكري بالمستوى المعيشي	
رمز المشارك : ………………………………………	
المحور الأول : أثر العلاج على الأعمال اليومية والحياة الاجتماعية	
Q1	علاجي الحالي للسكري يؤثر على عملي ونشاطاتي اليومية.
	1 2 3 4 5 6 7
Q2	علاجي الحالي للسكري يحد من نطاق نشاطاتي اليومية.
	1 2 3 4 5 6 7
Q3	من الصعب إيجاد الوقت أو المكان المناسب لتعاطي علاجي للسكري.
	1 2 3 4 5 6 7
Q4	علاجي الحالي للسكري يحد من نشاطاتي الجماعية والشخصية مع المعارف والأصدقاء.
	1 2 3 4 5 6 7
Q5	أجد مشقة في الاستيقاظ بشكل يومي كل صباح لتعاطي علاج السكري.
	1 2 3 4 5 6 7
Q6	في إطار علاجي الحالي للسكري، فإن أوقات الطعام المحددة تسبب لي مشقة.
	1 2 3 4 5 6 7
Q7	أجد صعوبة في الأكل في المطاعم في إطار علاجي الحالي للسكري.
	1 2 3 4 5 6 7
Q8	أشعر بأن علاجي الحالي للسكري يسلب مني متعة الأكل.
	1 2 3 4 5 6 7
Q9	مع علاجي الحالي للسكر، أجد بأنه من الصعب على إشباع شهيتي.
	1 2 3 4 5 6 7
Q10	الوقت والطاقة الذي يحتاجه متابعة علاجي للسكري يسبب مشقة بالنسبة لي.
	1 2 3 4 5 6 7
Q11	أجد نفسي متأرقاً حيال إدارتي للوقت مع متابعتي لعلاجي للسكري.
	1 2 3 4 5 6 7
Q12	أجد آلام بسبب علاجي للسكري تسبب لي عدم الارتياح.
	1 2 3 4 5 6 7
Q13	أثار التلبك المعوي (الغثيان، الغازات، الإسهال، آلام معوية) تسبب لي عدم الارتياح بسبب تعاطي علاج السكري.
	1 2 3 4 5 6 7
المحور الثاني : التوتر وعدم الرضى بالعلاج	
Q14	مع علاجي الحالي للسكري أشعر بعدم ارتياح بسبب زيادة الوزن.
	1 2 3 4 5 6 7
Q15	أجد آثار متعبة بسبب ارتفاع السكر بالدم.
	1 2 3 4 5 6 7
Q16	أنا قلق بشأن ارتفاع السكر بالدم.
	1 2 3 4 5 6 7
Q17	أنا غير راضي عن مستوى السكر في الدم المضطرب بين الانخفاض والارتفاع.
	1 2 3 4 5 6 7
Q18	أقلق بأن تزداد المضاعفات حدةً بسبب علاجي الحالي للسكري.
	1 2 3 4 5 6 7
Q19	يصيبني القلق عند التفكير بأسباب الحياة بسبب علاجي الحالي للسكري.
	1 2 3 4 5 6 7
Q20	أجد بأنه من غير المحتمل التصور بأنه وبالرغم من متابعتي لعلاج السكري قد لا أشفى من مرض السكري.
	1 2 3 4 5 6 7
Q21	أشعر بقلق بأنه ومع متابعتي لأخذ العلاج للسكري سيضمحل أثر العلاج مع الوقت.
	1 2 3 4 5 6 7
المحور الثالث : انخفاض السكر بالدم	
Q22	أقلق بشأن انخفاض سكر الدم بسبب علاج السكر الذي أتعاطاه الآن.
	1 2 3 4 5 6 7
Q23	أشعر بالخوف بسبب انخفاض سكر الدم.
	1 2 3 4 5 6 7
Q24	أشعر أحياناً بعدم ارتياح بسبب انخفاض سكر الدم.
	1 2 3 4 5 6 7
Q25	آثار انخفاض سكر الدم غير مريحة لي.
	1 2 3 4 5 6 7
المحور الرابع : الرضى عن العلاج	
Q26	بشكل عام، أنا راضي عن مستوى السيطرة على سكر الدم.
	1 2 3 4 5 6 7
Q27	أشعر بثقة بأن علاجي للسكر الحالي سيسيطر على مستوى سكر الدم.
	1 2 3 4 5 6 7
Q28	أشعر بأمل بشأن المستقبل بسبب التزامي بعلاجي للسكري.
	1 2 3 4 5 6 7
Q29	بالنسبة للعلاج من السكر، أنا راضي عن أساليب العلاج من مرض السكري.
	1 2 3 4 5 6 7
